# The impact of maternal smoking during pregnancy on depressive and anxiety behaviors in children: the Norwegian Mother and Child Cohort Study

**DOI:** 10.1186/s12916-014-0257-4

**Published:** 2015-02-03

**Authors:** Steven Moylan, Kristin Gustavson, Simon Øverland, Evalill Bølstad Karevold, Felice N Jacka, Julie A Pasco, Michael Berk

**Affiliations:** School of Medicine, Deakin University, Geelong, Victoria 3220 Australia; Division of Mental Health, Norwegian Institute of Public Health, Oslo, NO-0403 Norway; Faculty of Psychology, University of Bergen, Bergen, N-5020 Norway; Department of Psychology, University of Oslo, Oslo, NO-0316 Norway; NorthWest Academic Centre, Department of Medicine, The University of Melbourne, St Albans, Victoria 3021 Australia; Department of Psychiatry, Orygen Research Centre, and the Florey Institute for Neuroscience Mental Health, The University of Melbourne, Melbourne, Parkville Victoria 3052 Australia; Barwon Health, Geelong, Victoria 3220 Australia

**Keywords:** Anxiety, Depression, Cigarette smoking, Pregnancy, Obstetrics, Psychiatry

## Abstract

**Background:**

Maternal smoking during pregnancy (MSDP) is associated with multiple adverse childhood outcomes including externalizing behaviors. However, the association between MSDP and internalizing (anxiety and depressive) behaviors in offspring has received less investigation. We aimed to assess the association between MSDP and childhood internalizing (anxiety and depressive) behaviors in a very large, well-characterized cohort study.

**Methods:**

We assessed the association between MSDP and internalizing behaviors in offspring utilizing information drawn from 90,040 mother-child pairs enrolled in the Norwegian Mother and Child Cohort Study. Mothers reported smoking information, including status and frequency of smoking, twice during pregnancy. Mothers also reported their child’s internalizing behaviors at 18 months, 36 months, and 5 years. Associations between MSDP and childhood internalizing behaviors, including dose-response and timing of smoking in pregnancy, were assessed at each time point.

**Results:**

MSDP was associated with increased internalizing behaviors when offspring were aged 18 months (B = 0.11, *P* <0.001) and 36 months (B = 0.06, *P* <0.01), adjusting for numerous potential confounders. Higher rates of smoking (e.g., >20 cigarettes per day) were associated with higher levels of internalizing behaviors. Maternal smoking during early pregnancy appeared to be the critical period for exposure.

**Conclusions:**

We found evidence supporting a potential role for MSDP in increasing internalizing (anxiety and depressive) behaviors in offspring. We also found evidence supportive of a possible causal relationship, including dose-dependency and support for a predominant role of early pregnancy exposure. Further investigation utilizing genetically informed designs are warranted to assess this association.

## Background

Maternal smoking during pregnancy (MSDP) is associated with numerous adverse outcomes in children. These occur in physical, cognitive, and behavioral domains, including stillbirth [1], lowered birth weight [2], childhood asthma [3], obesity [4], intelligence [5], hyperactivity, impulsivity, and conduct problems [6-10]. Hypotheses explaining these associations include direct causation (e.g., cigarette components directly damaging developing fetal structures and physiological systems) and shared vulnerability [11-13] (e.g., genetic and/or environmental factors increasing rates of MSDP and childhood outcomes). In the realm of childhood behavioral outcomes, consistent findings from multiple prospective observational studies [14], controlling for plausible confounders, support an argument for a direct causal relationship (e.g., via physiological effects) rather than one solely underpinned by shared vulnerability. This is complimented by animal and human research [15] demonstrating that MSDP can disrupt neurodevelopment via effects on maturing neurotransmitter systems and brain architecture in regions associated with stress and mood regulation (e.g., the hippocampus [16] and somatosensory cortex [17]).

Despite these findings, however, debate continues regarding whether these associations represent causal relationships [18-20]. As noted by several authors [19,21], MSDP is associated with numerous social and environmental factors (e.g., teenage motherhood, lower maternal education [11], increased single motherhood [22]) that influence childhood outcomes. In addition, genes that influence the likelihood of MSDP [23] may also affect childhood outcomes through maternal-child genetic inheritance. For this reason, studies utilizing quasi-experimental designs [19] (e.g., siblings with discordant exposures [24], children from *in vitro* fertilization [25]), predominantly investigating MSDP-childhood externalizing behavior associations, have been undertaken in an attempt to control for unmeasured genetic and environmental confounders. These studies have generally demonstrated attenuation of previously observed MSDP-childhood externalizing behavior associations [11,21,26], though exceptions exist [27]. Although these approaches have advantages over traditional observational methods [19] and should be pursued further [19,28], they are not without problems [29]. For example, most studies utilizing discordant sibling and *in vitro* fertilization analysis have fewer participants, reducing power to detect true associations. Additionally, discordant samples are a highly selected group [29], with factors that underpin maternal behavioral change also potentially impacting on childhood outcomes. Further, siblings share on average only 50% of alleles, and therefore differential effects, even ones protective of adverse behavioral outcomes, could be influenced by variations in the other 50% of the inherited alleles. It is therefore unlikely that any single study design will be completely capable of determining causal inference, and a range of approaches will be required. In contrast to the effort expended in exploring the MSDP-childhood externalizing behavior association, fewer studies have explored the association between MSDP and childhood internalizing (anxiety and depressive) behaviors. Those that have investigated this association report both positive and null results [30-34]. Limitations of these studies, such as small sample sizes, limited controls for potential confounders, and variable reporting of smoking, amongst other issues, may have contributed to these inconsistencies.

Anxiety and depressive disorders represent a significant contribution to the global burden of disease [35]. In contrast to many potential risk factors associated with childhood outcomes (e.g., genetics), MSDP is potentially preventable. For this reason, a greater understanding of the MSDP-childhood internalizing behavior association is very relevant for public health. We therefore aimed to build on previous observational studies to investigate the MSDP-childhood internalizing behavior association in a very large and well-characterized prospective study. We tested hypotheses that i) there would be a dose-response effect, such that higher cigarette consumption would be associated with higher internalizing behaviors, and ii) that early pregnancy, as a phase of rapid neurodevelopment, would be a sensitive period. If such an association existed in a large observational study, further investigation via a genetically informed design would have significant merit.

## Methods

### Study design and participants

The Norwegian Mother and Child Cohort Study (MoBa) is a prospective population-based study that aimed to recruit all women who gave birth in Norway between 1999 and 2009 [36]. The sampling frame consisted of women who attended for routine ultrasound examination at approximately 17 weeks of gestation. Participation rates were 38.7% of those invited. For this study, information was available on 107,379 children (51.2% boys) who were born to 89,962 participating mothers. Only one child was included when the mother had twins or triplets to reduce problems with dependency between observations. The twin/triplet registered first in the data files was included in the analyses. Of these respondents, we were able to utilize information from 90,040 mother-child pairs where smoking information was available. Mothers completed detailed questionnaires on their health and social status at gestational weeks 17 (questionnaire 1) and 30 (questionnaire 3), and questionnaires on the health and development of their children at multiple time points after birth, including at ages 18 months (questionnaire 5), 36 months (questionnaire 6), and 5 years (questionnaire 7). Of those who agreed to participate in MoBa, the response rates were 94.9% for questionnaire 1 (early pregnancy), 91.0% for questionnaire 3 (late pregnancy), 72.5% for questionnaire 5 (18 month follow-up), 58.5% for questionnaire 6 (36 month follow-up), and 53.0% for questionnaire 7 (5 year follow-up). All participants provided written informed consent, and ethics approval was obtained from The Regional Committee for Medical Research Ethics in South-Eastern Norway.

### Exposure: maternal smoking

Mothers self-categorized their current and past smoking status (daily, occasional, or non-smoking) and also reported the number of cigarettes they smoked per day or week, at gestational weeks 17 (‘early pregnancy’) and 30 (‘late pregnancy’). We subsequently dichotomized MSDP (smoking/non-smoking). Mothers who reported smoking any cigarettes per day or week were considered smokers even if they self-categorized as non-smokers. Mothers who provided smoking data from early and late pregnancy were categorized into four groups: smoking only in early pregnancy, smoking only in late pregnancy, smoking throughout pregnancy, and no smoking in pregnancy. We calculated a smoking frequency for all women in early pregnancy by using self-reported daily cigarette consumption or, where not available, dividing self-reported weekly cigarette consumption by seven. These data were used to categorize early pregnancy smokers into four groups: no smoking in early pregnancy, 1 to 9 cigarettes per day, 10 to 19 cigarettes per day, and 20+ cigarettes per day. For the final analysis, we also incorporated data from questionnaire 1 to create a dichotomous variable (yes/no) coding whether women had smoked in previous pregnancies.

### Outcome: childhood anxiety and depressive (internalizing) behaviors

Mothers reported their child’s internalizing behaviors by answering questions taken from a condensed 25 question version of The Childhood Behavior Checklist (CBCL) [37] at ages 18 months, 36 months, and 5 years.

At 18 months, internalizing behaviors were assessed using five items from the internalizing scale of CBCL. Mothers rated on a 1 to 3 scale (1: ‘Not true’, 2: ‘Somewhat or sometimes true’, 3: ‘Very true or often true’) the extent to which their child’s behavior was consistent with the following statements over the previous 2 months:

1. “Clings to adults or too dependent”
2. “Gets too upset when separated from parents”
3. “Too fearful or anxious”
4. “Disturbed by any change in routine”
5. “Does not eat well”

The first three items are from the anxious/depressed subscale of the internalizing scale, the fourth from the emotionally reactive subscale, and the fifth from the somatic complaints subscale. A mean score of the five items was computed to represent overall internalizing behaviors.

At 36 months, internalizing behaviors were assessed using nine items from the CBCL, including the five items used at 18 months plus four additional items:

1. “Constipated doesn’t move bowels”
2. “Stomach aches or cramps (without medical cause)”
3. “Vomiting, throwing up (without medical cause)”
4. “Sudden changes in moods or feelings”

The first three items are from the somatic complaints subscale and the fourth from the emotionally reactive subscale. Again, each item was rated 1 to 3, with a mean score of the nine items computed to represent overall internalizing behaviors.

At 5 years, internalizing behaviors were assessed using 11 items from the CBCL, including the five items used at 18 months plus six additional items:

1. “Feelings are easily hurt”
2. “Nervous, high-strung, or tense”
3. “Self-conscious or easily embarrassed”
4. “Unhappy, sad, or depressed”
5. “Stomach aches or cramps (without medical cause)”
6. “Vomiting/throwing up (without medical cause)”

The first four items were from the anxious/depressed subscale, and last two from the somatic complaints subscale. Each item was rated 1 to 3 (1: ‘Never/rarely’, 2: ‘Sometimes’, 3: ‘Often/typical’) and a mean score of the 11 items was computed to represent overall internalizing behaviors. Mean inter-item correlations were used as estimates of internal consistency. Clark and Watson [38] argue that the mean inter-item correlation is a useful index of internal consistency for such scales, and recommend that this should be in the range 0.15 to 0.20 for broad constructs. Correlations measured 0.14, 0.13, and 0.16 at 18 months, 36 months, and 5 years, respectively, which were very close to this optimal range. Factor analysis suggested that scale items were best explained by one factor at each time point (results available on request). The CBCL possesses good predictive validity in the Norwegian population [39], with the Norwegian translation performed by Nøvik [39,40] used in this study.

### Covariates

We statistically controlled for a series of potential confounding variables, including paternal smoking, maternal education, maternal age, maternal depressive and anxiety symptoms, maternal alcohol consumption, parity, gestational age at birth, and smoking in previous pregnancies. Information on these covariates was obtained from questionnaire 1.

Paternal smoking status was assessed from the fathers’ questionnaire undertaken during pregnancy and dichotomized. Where fathers had not participated, their smoking status was obtained from the mother’s report of their smoking status. Maternal education, utilized as a proxy for socioeconomic status, was assessed by self-report on a 5-point scale (1: ≤9 years of schooling; 2: 1 to 2 years of high school; 3: technical high school or junior college education; 4: 1 to 4 years in college or university; 5: >4 years in college or university). Maternal age in early pregnancy was calculated from information provided by the medical birth register. Maternal depressive and anxiety symptoms were assessed in early pregnancy by self-report of the Hopkins Symptom Checklist 5 (HSCL-5). This scale is a short version of the Hopkins Symptom Checklist 25 (HSCL-25), consisting of five items (“Feeling fearful”, “Nervousness or shakiness inside”, “Feeling hopeless about the future”, “Feeling blue”, and “Worrying too much about things”) rated on a 4-point scale (from not bothered to very bothered). The HSCL-5 correlates strongly with the HSCL-25 [41,42]. Cronbach’s alpha in the current sample was 0.80. Maternal alcohol consumption was assessed on a 7-point scale ranging from never to approximately 6 to 7 times a week. Information on parity, child’s gender, and gestational age at birth was obtained from the Norwegian birth register. Mothers provided information on previous pregnancies in questionnaire 1, including the number of previous pregnancies and individual pregnancy details including birth status, breastfeeding status, weight gain during pregnancy, and a dichotomous tick box for “Smoked during pregnancy”.

We performed correlations of all potential confounding factors to assess their relationship between exposures (MSDP characteristics) and outcomes (internalizing behaviors). Due to rules regarding the publication of covariate effects derived from MoBa data we are unable to report correlation data in its entirety. However, of our potential confounding factors, only child gender did not display associations between exposure and outcome and hence was not included in adjusted models.

### Statistical analyses

All statistical analyses were performed in SPSS version 20. Only subjects without missing data points were included in each individual analysis. The internalizing scale was log-transformed due to a non-normal distribution (right or positively skewed). A right skewed distribution meant that most respondents reported low levels of internalizing problems – as one would expect in a community sample. The log-transformed scale was then standardized for ease of interpretation. Standardizing allowed comparison of results across analyses using internalizing behavior at different time points as outcomes. ANOVA was performed to compare level of internalizing symptoms among children of mothers who did not smoke to the level of such symptoms among children of the different groups of smokers (e.g., early in pregnancy, late in pregnancy, and throughout pregnancy). The ANOVAs were performed using generalized estimating equations (GEE) with the “sandwich estimator” because of violations of the assumptions of linear models about homogeneity of variance and independence between observations. This estimator provides robust standard errors, especially when N and the number of independent observations are large [43]. We used an unstructured correlation matrix, as this does not put any *a priori* restrictions on the modelling of these correlations, while an independent correlation matrix treats related observations (e.g., siblings) as not more correlated than other observations. Both of these are possible models for siblings’ data and were checked. The one with the best fit to the data was selected – the independent correlation matrix. We interpreted that correlations between siblings’ data were not found to be a major concern in our analyses. GEE was still preferred over usual ANOVA as GEE allows using models with unequal variances in different groups.

Basic descriptive statistics and Pearson correlations of study variables were performed. We used ANOVA models to examine mean differences between MSDP status and childhood internalizing behaviors at 18 months, 36 months, and 5 years. Model A was unadjusted, Model B was adjusted for paternal smoking, maternal alcohol consumption, maternal depressive and anxiety symptoms, maternal age, maternal education level, parity, and gestational age at birth, and Model C further adjusted for retrospective reporting of smoking in previous pregnancies. For each model and subsequent analysis, only participants reporting all required data were included.

We hypothesized that the impact of smoking on internalizing behaviors would be largest in early pregnancy, as this is a time of rapid neurodevelopment [44]. Therefore, we tested the association between timing of MSDP (early only, late only, throughout, or no smoking) on internalizing behaviors at 18 months, 36 months, and 5 years. We then compared the effect size of smoking only in early pregnancy and smoking only in late pregnancy with the effect size of smoking throughout pregnancy at each time point. We hypothesized that if the effect size of smoking in a particular stage of pregnancy (e.g., early or late) was significantly weaker than the effect size of smoking throughout pregnancy, then this would suggest exposure to MSDP at this stage contributed less to internalizing behavior expression than exposure in the alternative stage of pregnancy. We undertook significance testing between comparing the effects of early pregnancy smoking and late pregnancy smoking with smoking throughout pregnancy by using the standard error (SE) of the difference as advised by Cohen et al. [45]. This procedure involves calculating the SE of the difference between effect sizes (SE_diff_ = sqrt (SE_B1_^2^ + SE_B2_^2^)) and then dividing the difference between effects by this SE_diff_ to obtain z-scores for the differences.

Further, we tested whether a dose-response relationship existed between daily maternal cigarette consumption in early pregnancy and internalizing behaviors in children at 18 months, 36 months, and 5 years. Linear models comparing three categories of smoking frequency (20+ per day, 10 to 19 per day, 1 to 9 per day) were compared to non-smokers and adjusted for Model B covariates.

Finally, we undertook a subsample analysis of mother-child pairs where information was available regarding smoking in previous pregnancies (n = 65,439) to test MSDP-childhood internalizing behavior associations at ages 18 months, 36 months, and 5 years adjusting for Model B covariates plus smoking in previous pregnancies (Model C).

As discussed in the introduction, MSDP and childhood behavioral outcomes are associated with various genetic and environmental factors that are challenging to measure in observational studies. If uncontrolled, these factors may contribute to spurious associations being observed between MSDP and childhood behaviors. MSDP could influence childhood internalizing behavior expression in two ways. First, MSDP could influence internalizing behavior expression through direct effects on the developing fetus. Second, MSDP and internalizing behaviors could be indirectly linked through a series of shared genetic and environmental factors unrelated to the direct smoking effects. In contrast to MSDP, smoking in a past pregnancy can only be associated with a current child’s internalizing behaviors through effects mediated by shared genetic and environmental factors (as no direct smoking effects are possible). Therefore, given a portion of these indirect factors (e.g., maternal genetics, maternal education level) will be consistent between past and current pregnancies, controlling for this variable is likely to capture some of the indirect effects influencing the MSDP-internalizing behavior association.

Although limitations exist for this approach, by controlling for as many indirect effects as possible, we aimed to approximate the direct impact of MSDP (e.g., by disruption of normal neurodevelopment) on the current child’s internalizing behaviors.

## Results

### Descriptive statistics

**Figure 1:**
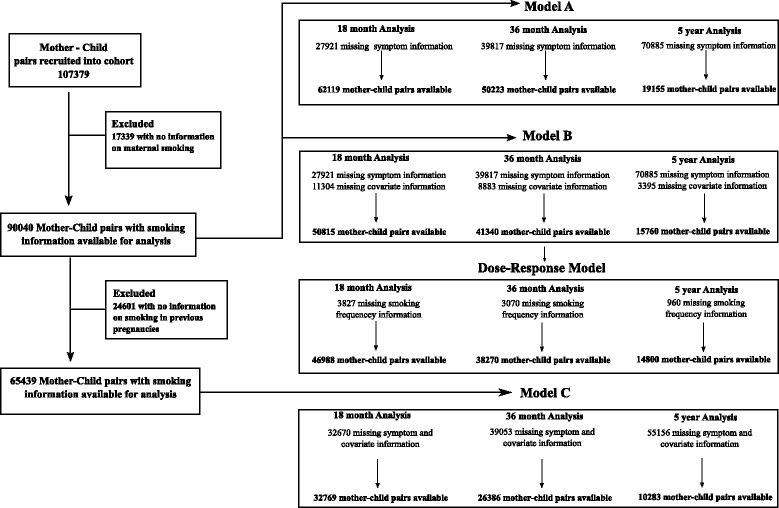
**Study profile.** Figure 1 details the data available for each individual analysis performed in the study. Data from a total of 107,379 mother-child pairs was available in the Norwegian Mother and Child Cohort Study at the time of this study, of which 90,040 provided information about maternal smoking during pregnancy. For each model described, the number of mother-child pairs excluded due to missing internalizing symptom information is listed first, followed by the total number of mother-child pairs available for the specific analysis at each time point. Model A represents the unadjusted model. Model B represents the main confounder model adjusted for covariates paternal smoking, maternal alcohol consumption, maternal depressive and anxiety symptoms, maternal age, maternal education level, parity, and gestational age at birth. Dose-response analysis includes controls for Model B covariates. Model C represents the main confounder model in a subsample of mothers who provided information on smoking in past pregnancies. Analyses in Model C include Model B covariates plus adjustment for smoking in previous pregnancies.

The sample sizes available for the different analyses are shown in Figure 1. Mean maternal age at birth was 29.8 (standard deviation (SD), 4.6) years and mean gestational age at birth was 39.3 (SD, 2.26) weeks. Smoking information was reported in 90,040 pregnancies, with maternal smoking in early pregnancy present in 8,418 pregnancies (9.3%). Mean maternal depression and anxiety in early pregnancy on the HSCL-5 was 1.26 (SD, 0.40). Information on internalizing behaviors was available for 69,946 children at 18 months, 57,143 children at 36 months, and 19,778 children at 5 years. Mean internalizing behaviors were 1.27 (SD, 0.25) at 18 months, 1.25 (SD, 0.22) at 36 months, and 1.16 (0.19, SD) at 5 years. Descriptive statistics of exposures, outcomes, and covariates are included in Table 1. Missing information on questionnaires lead to exclusion of 5,274 mother-child pairs at 18 months, 407 mother-child pairs at 36 months, and 95 mother-child pairs at 5 years. Compared to eligible participants, children excluded due to missing data or being lost to follow-up had mothers who were more likely to be younger, less well educated, had higher rates of depression, higher parity, and were smokers in early pregnancy (results available on request).

**Table 1:** **Descriptive statistics of exposures, outcomes, and included covariates**

	**Mean (SD)**
**Maternal age (years)**	29.8 (4.6)
**Gestational age at birth (weeks)**	39.34 (2.26)
**Maternal depression and anxiety in early pregnancy (range 1 to 4)**	1.26 (0.40)
**Maternal education**	**N (% total)**
≤9 years education	2,782 (2.6%)
1 to 2 years high schooling	4,944 (4.6%)
3 years schooling	26,854 (25.0%)
1 to 4 college/university	39,388 (36.7%)
>4 years college/university	22,579 (21.0%)
Missing	10,832 (10.1%)
**Maternal alcohol consumption**	**N (% total)**
Never	74,702 (69.6%)
<1 episode per month	8,615 (8.0%)
1 to 3 episodes per month	1,995 (1.9%)
<1 episode per week	429 (0.4%)
2 to 3 episodes per week	61 (0.1%)
4 to 5 episodes per week	7 (0.0%)
6 to 7 episodes per week	15 (0.0%)
Missing data	21,555 (20.1%)
**Maternal parity**	**N (% total)**
0	47,515 (44.2%)
1	38,188 (35.6%)
2	16,443 (15.3%)
3	3,610 (3.4%)
4+	1,132 (1.1%)
Missing data	492 (0.5%)
**Paternal smoking (early pregnancy)**	**N (% total)**
Yes	25,039 (23.3%)
No	76,079 (70.9%)
Missing data	6,261 (5.8%)
**Internalizing symptoms (range 1 to 3)**	**Mean (SD)**
18 months (n = 69,946)	1.27 (0.25)
36 months (n = 57,143)	1.25 (0.22)
5 years (n = 19,778)	1.16 (0.19)

Internalizing symptoms are drawn from maternal report from a condensed version of the CBCL. Maternal depression and anxiety symptoms are drawn from the HSCL-5.

### Linear associations

Linear associations were noted between increased maternal alcohol consumption, maternal depressive and anxiety symptoms, parity and paternal smoking, and increased maternal smoking in early and late pregnancy. Increasing maternal age, maternal education, and gestational age at birth were correlated with decreased maternal smoking in early and late pregnancy. Child gender was not associated with maternal smoking. Internalizing behaviors at 18 months, 36 months, and 5 years were strongly positively correlated. Linear associations were noted between increased internalizing behaviors at all-time points and increased paternal smoking, maternal depressive and anxiety symptoms, and child gender (boys). Increasing maternal age, maternal education, gestational age, and parity were associated with decreased internalizing behaviors at all-time points. Linear associations of relatively small effect size were observed between MDSP and internalizing symptoms at all time points (Table 2).

**Table 2:** **Correlations between maternal smoking, smoking in previous pregnancies, and internalizing behaviors in children at 18 months, 36 months, and 5 years**

	**Maternal smoking early pregnancy (dichotomized)**	**Maternal smoking early pregnancy (cigarettes per day)**	**Maternal smoking late pregnancy (dichotomized)**	**Maternal smoking in previous pregnancy**	**Internalizing behaviors (18 months)**	**Internalizing behaviors (36 months)**	**Internalizing behaviors (5 years)**
**Maternal smoking early pregnancy (dichotomized)**	1						
**Maternal smoking early pregnancy (cigarettes per day)**	n/a	1					
**Maternal smoking late pregnancy (dichotomized)**	0.840**	0.822**	1				
**Maternal smoking in previous pregnancy**	0.534**	0.519**	0.519**	1			
**Internalizing behaviors (18 months)**	0.065**	0.065**	0.057**	0.067**	1		
**Internalizing behaviors (36 months)**	0.051**	0.050**	0.043**	0.073**	0.354**	1	
**Internalizing behaviors (5 years)**	0.031**	0.039**	0.029**	0.051**	0.272**	0.404**	1

Internalizing behaviors have been log transformed and standardized. Correlations are Pearson Coefficients. **P <0.001.

### Associations between MSDP, timing of MSDP, and childhood internalizing symptoms

**Figure 2:**
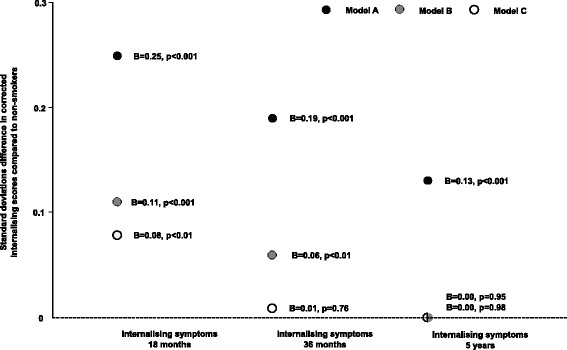
**Associations between maternal smoking and internalizing behaviors in children at 18 months, 36 months, and 5 years adjusting for Model A, Model B, and Model C covariates.** This figure displays the individual B coefficients for associations between maternal smoking during pregnancy and internalizing behaviors at different time points for Models A, B, and C. Associations were discovered at all time points for Model A (unadjusted), and at 18 months and 36 months for Model B (main confounder model). After controlling for smoking in past pregnancies only associations at age 18 months remained significant. The effect sizes decreased as children grew older. Non-smoking mother-child pairs are the reference group. Internalizing behaviors have been log transformed and standardized. B coefficients should be interpreted as units of standard deviation difference in corrected internalizing behaviors between smokers and non-smokers. * Model A: Unadjusted model. † Model B: Main confounder model adjusted for paternal smoking, maternal alcohol consumption, maternal depressive and anxiety symptoms, maternal age, maternal education level, parity, and gestational age at birth. ‡ Model C: Main confounder model in subsample of mothers, Model B plus adjustment for smoking in previous pregnancies. For Model A, ratio of smokers to non-smokers was 4,663/57,456, 3,728/46,495 and 1,167/17,988 for analysis at time points 18 months, 36 months, and 5 years, respectively. For Model B, the ratio of smokers to non-smokers was 3,829/46,986, 3,072/38,268, and 960/14,800 for analysis at time points 18 months, 36 months, and 5 years, respectively. For Model C, the ratio of smokers to non-smokers was 2,717/30,052, 2,157/24,229, and 663/9,620 for analysis at time points 18 months, 36 months, and 5 years, respectively.

Maternal smoking in early pregnancy and throughout the entire pregnancy was associated with increased internalizing behaviors in children at 18 months, 36 months, and 5 years unadjusted for confounding variables (Model A, Table 3). Maternal smoking in late pregnancy only was associated with increased internalizing behaviors at 18 months but not at later time points. In our analysis of timing of smoking during pregnancy, we found insufficient evidence to conclude any differences between mothers who smoked only in early pregnancy and those who smoked throughout pregnancy on children’s internalizing behaviors observed at 18 months (*P* ≥0.05). However, the observed effect size of association of smoking only in late pregnancy was smaller than the observed effect size of association of smoking throughout pregnancy (*P* <0.01), possibly indicating maternal smoking in early pregnancy underpins this effect. No significant differences in associations were observed in children at 36 months and 5 years. After adjusting for Model B covariates, maternal smoking in early pregnancy remained associated with increased internalizing behaviors at 18 months and 36 months, but not 5 years. As observed for Model A, the relative magnitude of the association between maternal smoking and subsequent internalizing behaviors after adjustment reduced as children aged (Figure 2).

**Table 3:** **Associations between timing of maternal smoking and internalizing behaviors in children at 18 months, 36 months, and 5 years**

	**Dependent variable**		
	**Internalizing behaviors (18 months; n = 62,119)**	**Internalizing behaviors (36 months; n = 50,223)**	**Internalizing behaviors (5 years; n = 19,155)**
**Maternal non-smokers**	Reference	Reference	Reference
**Maternal smoking in early pregnancy only**	B = 0.20 (SE, 0.04), *P* <0.001	B = 0.17 (SE, 0.04), *P* <0.001	B = 0.15 (SE, 0.07), *P* = 0.03
**Maternal smoking in late pregnancy**	B = 0.11 (SE, 0.05), *P* = 0.04	B = 0.10 (SE, 0.06), *P* = 0.09	B = 0.21 (SE, 0.11), *P* = 0.06
**Maternal smoking throughout pregnancy**	B = 0.19 (SE, 0.02), *P* <0.001	B = 0.19 (SE, 0.02), *P* <0.001	B = 0.11 (SE, 0.04), *P* <0.01

Maternal non-smokers are the reference group, i.e., each of the three smoking groups is compared to this group in the analyses. Internalizing behaviors have been log transformed and standardized. B coefficients should be interpreted as units of standard deviation difference between each smoking group and non-smokers in the log-transformed scale for internalizing behaviors.

SE, Standard error.

### Dose-response relationship between MSDP and childhood internalizing symptoms

**Figure 3:**
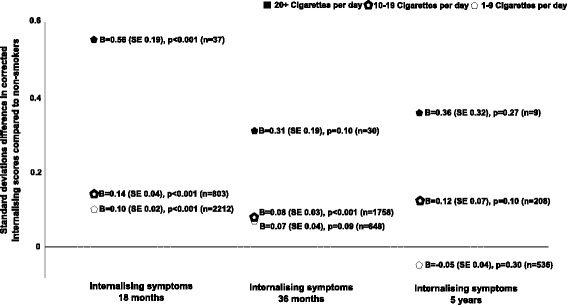
**Associations between different daily maternal cigarette consumption and internalizing behaviors in children at 18 months, 36 months, and 5 years.** This figure displays the individual B coefficients for associations between different daily rates of maternal smoking during prgnancy and internalizing behaviors at different time points adjusted for Model B covariates. Associations were discovered for all rates of smoking at 18 months. Non-smoking mother-child pairs are the reference group. Internalizing behaviors have been log transformed and standardized. B coefficients should be interpreted as units of standard deviation difference in corrected internalizing behaviors between smokers and non-smokers. All analyses adjusted for Model B covariates paternal smoking, maternal alcohol consumption, maternal depressive and anxiety symptoms, maternal age, maternal education level, parity, and gestational age at birth. Reported n’s are numbers of smokers at each time point in that smoking category. N’s for maternal non-smokers were 46,986, 38,268, and 14,800 at time points 18 months, 36 months, and 5 years, respectively.

A dose-response relationship was observed between frequency of maternal smoking in early pregnancy and childhood internalizing behaviors at 18 months, adjusting for Model B covariates (Figure 3). Smoking 20+ cigarettes daily conferred a much larger effect (B = 0.56, SE = 0.19, *P* <0.01) than smoking 10 to 19 cigarettes (B = 0.14, SE = 0.04, *P* <0.001) or 1 to 9 cigarettes (B = 0.10, SE = 0.20, *P* <0.001), when compared to not smoking. These results suggest that children of mothers who smoked 20+ cigarettes during pregnancy displayed an average of 0.56 SD higher levels of internalizing symptoms at age 18 months than children of non-smoking mothers, on the log-transformed scale. Similar disparities in observed associations with different daily smoking rates were present at 36 months and 5 years, but failed to reach significance.

### Associations between MSDP and childhood internalizing symptoms, controlling for smoking in past pregnancies

In a subsample of women who provided information about previous pregnancies (n = 65,439), smoking in previous pregnancies was associated with increased current childhood internalizing behaviors at 18 months (B = 0.04, *P* <0.05) and 36 months (B = 0.10, *P* <0.01), but not at 5 years. Adjusting for Model B covariates plus smoking in previous pregnancies (Model C) showed that maternal smoking in early pregnancy was associated with increased internalizing behaviors in children at 18 months, but not at 36 months and 5 years, when compared to non-smokers.

## Discussion

In this large, well-characterized prospective study, MSDP was associated with increased internalizing behaviors in children, adjusting for a broad range of possible confounders. A dose-dependent effect of MSDP was evident. There was a larger adverse impact of smoking in early pregnancy than in late pregnancy. Finally, smoking in previous pregnancies was associated with increased internalizing behaviors in subsequently born children at age 18 months, suggesting this variable may capture some portion of residual confounding (e.g., genetic risk, environmental influences) not included in models. Controlling for this variable did not, however, eliminate the observed association.

Our data builds significantly on previous observational literature that has demonstrated inconsistent results. For example, analysis of 2,758 mother-child pairs from the RAINE study revealed that children displayed higher internalizing behaviors between ages 2 and 14 if their mother failed to quit smoking by week 18 of pregnancy (OR 1.55, *P* = 0.006), also after controlling for a range of potential confounders [34]. Children of women who quit prior to gestational week 18, irrespective of their previous smoking levels, did not display different levels of internalizing behaviors than children of non-smoking mothers. These results contrast with outcomes from two large cohorts, where adjustment for confounders eliminated the association. In assessment of The Generation R study (n = 4,680), effects of MSDP on childhood internalizing behaviors at 18 months were completely confounded by adjustment for parental educational level, family income, national origin, parental psychopathology, and child gender [46]. In the Avon Longitudinal study (n = 4,394), MSDP was not associated with increased internalizing behaviors in children aged 4 years, after controlling for a range of potential confounders including socioeconomic status, parental psychopathology, and alcohol consumption [32].

The inconsistency in published studies may reflect power issues, varying ages of assessment, and differences in included confounders or populations assessed. For example, the overall risk of behavioral problems in the Generation R Study sample was lower than that of the normal Dutch population, potentially limiting capacity to find small associations [46]. Our study found the largest impact of MSDP appeared in children aged 18 months, with magnitude of associations diminishing over time. This observation may reflect an evolution from a greater impact of genetic and intrauterine factors immediately after birth, towards an increasing impact of prevailing psychosocial factors on internalizing behavior expression as children grow up. These data are concordant with studies demonstrating positive associations between MSDP and internalizing behaviors when assessing younger (e.g., 1 to 3 years) [33,34] but not older (e.g., age >4) [9,32,47] children, although exceptions exist [30,46,48].

Most toxins display a dose-dependent relationship. Our finding of the greatest impact on internalizing behaviors occurring in mothers who smoked 20+ cigarettes per day is therefore concordant with expected patterns. Our data additionally demonstrated the largest effect on childhood internalizing behaviors in those who smoked during early rather than late pregnancy. These findings are consistent with animal models that demonstrate prenatal nicotine exposure is associated with long term increases in anxiety behaviors [49]. It is hypothesized that increased anxiety behaviors observed in animals exposed to MSDP may at least partially relate to nicotine and other cigarette smoke components directly interfering with normal neurodevelopmental processes *in utero* [16,50]. Germane to first trimester effects of MSDP, nicotinic acetylcholine receptors are expressed very early (prior to neurulation) in gestation and serve a critical role in facilitating key aspects of neurodevelopment, including neurogenesis, planned apoptosis, and axonal and synaptic growth [16]. Abnormal activation of these receptors via exogenous nicotine has been demonstrated to interfere with these key neurodevelopmental processes, including in brain regions associated with mood and anxiety control [17,51]. These changes appear to be associated with later behavioral problems in animal models, including increased anxiety states [52], but it is not yet known if this applies to humans [53].

Despite this, whether our results represent a causal relationship is not yet determined. Causal assumptions from observational data are vulnerable to unmeasured confounding [18,26,28]. Although our observed associations sustained even after statistically (not experimentally) controlling for numerous important confounders, including maternal mental health, alcohol consumption, demographics, socioeconomic status, and paternal smoking, it remains possible that the dose-dependent MSDP-internalizing behavior association may still have resulted from unmeasured and uncontrolled genetic and environmental confounding (e.g., parental genetic factors) [19,21]. As genetic or adequate same-sibling data were not available in MoBA, we were unable to undertake a quasi-experimental design that could attempt to control for these genetic and environmental factors. Therefore, we attempted to address potential residual confounding by controlling the observed association for smoking in past pregnancies. We hypothesized that smoking in past pregnancies would act as a proxy that captures some residual genetic and environmental confounding as i) there was an observed positive association between smoking in a previous pregnancy and the current child’s internalizing behaviors after controlling for other included covariates, ii) genetic factors predisposing the mother to MSDP should be consistent across both pregnancies, and iii) many of the environmental factors influencing the likelihood of MSDP not captured through the measured confounding variables should be consistent across past and current pregnancies. Although this approach does not completely control for residual genetic and environmental confounding (e.g., parental genetic influences, changes to environmental circumstances between pregnancies), we believe this approach brings us one step closer to the true relationship. Including this variable into analysis, we found that the strength of association between MSDP in the current pregnancy and childhood internalizing behaviors at age 18 months decreased, but remained significant. Future studies utilizing aforementioned quasi-experimental designs in a large population would be of great benefit in further assessing the role of residual confounding.

### Limitations and directions for future research

Although this study has numerous strengths, including longitudinal follow-up of a very large and well-characterized cohort, the results should be interpreted in light of some limitations. The inability to confirm a causal association is common to all observational studies. In addition, our study was limited by maternal self-report of internalizing behaviors and smoking. Although previous investigations demonstrate that self-report of smoking correlates highly with measured cotinine levels in pregnancy [54], the lack of multiple informants for assessment of childhood internalizing symptoms (e.g., paternal, teacher, or clinician reports) to correlate the maternal reports is a limitation. We were unable to control for numerous paternal factors in addition to postnatal factors, including child-rearing proficiency, rates of breastfeeding [55], and exposure to environmental cigarette smoke that may have influenced expression of childhood internalizing behaviors. There was loss to follow-up throughout the study that may have distorted the findings, with children lost to attrition mainly having younger and less educated mothers, with higher rates of depression and anxiety symptoms, higher parity, and increased rates of smoking in early pregnancy. Monte Carlo simulations have shown that even though estimates of mean levels are sensitive to selective attrition, estimates of associations between variables are generally robust against selective attrition and that this is true even when non-response is related to unmeasured variables [56]. Although the MoBa study is a very large cohort and therefore confers substantial power to detect effects, for some analyses, such as those involving later ages of follow-up (e.g., 36 months and 5 years) and the dose dependence analysis (with relatively few mothers reporting certain behaviors, e.g., smoking >20 cigarettes per day), reduced availability of data may have reduced power to detect associations. MoBa utilized condensed versions of the CBCL scale to assess internalizing behaviors, which is a limitation, although factor analysis of the scales suggested that scale items were best explained by a single factor at each time point (results available on request). In addition, reporting of smoking in past pregnancies in Model C was retrospective, introducing the possibility of reporting bias. The MoBa cohort has a relatively low response rate that may represent a potential issue for generalizability to the Norwegian population, although the sample size remains substantial. In a comparison performed between data acquired in MoBa between 2000 and 2006 and data from the Norwegian Medical Birth Registry, maternal participants in MoBa were more likely to be older, less likely to be single, have lower parity, have lower rates of previous stillbirths, and less likely to suffer maternal asthma [57]. In addition, MoBa participants were more likely to be non-smokers, more likely to use folic acid, and less likely to suffer from gestational diabetes and placental abruption [57]. Although it is difficult to predict in what way these factors could bias our observations, we believe it is likely these factors (e.g., lower smoking rates) would be more likely to underestimate rather than overestimate associations.

## Conclusions

Overall, utilizing a very large mother-child cohort, our findings build significantly on the previous literature in demonstrating a population-wide association between MSDP and increased internalizing behaviors in children. Our study demonstrated a dose-dependent relationship and that the magnitude of observed association is greatest when mothers smoked in early pregnancy. The observed association sustained even after using smoking in past pregnancies as a proxy to control for some likely residual genetic and environmental confounding underpinning this relationship. These results suggest further studies, potentially utilizing quasi-experimental designs exploring the contribution of unmeasured genetic and environmental confounds as undertaken in other MSDP-childhood outcomes literature [19], are justified to further investigate the relationship between MSDP and internalizing behaviors in children. Consistent findings across differing study designs, in concert with the known deleterious impact of MSDP on other elements of childhood health [1,3,11], would provide further support for public health interventions aimed at reducing smoking in women of child-bearing age.

## Abbreviations

CBCL: The Childhood Behavior Checklist
GEE: Generalized estimating equations
HSCL-25: Hopkins Symptom Checklist 25
HSCL-5: Hopkins Symptom Checklist 5
MoBa: The Norwegian Mother and Child Cohort Study
MSDP: Maternal smoking during pregnancy
SD: Standard deviation
SE: Standard error

## References

[1] Flenady V, Koopmans L, Middleton P, Froen JF, Smith GC, Gibbons K, Coory M, Gordon A, Ellwood D, McIntyre HD, Fretts R, Ezzati M (2011). Major risk factors for stillbirth in high-income countries: a systematic review and meta-analysis. Lancet.

[2] Jaddoe VW, Troe EJ, Hofman A, Mackenbach JP, Moll HA, Steegers EA, Witteman JC (2008). Active and passive maternal smoking during pregnancy and the risks of low birthweight and preterm birth: the Generation R Study. Paediatr Perinat Epidemiol.

[3] Neuman A, Hohmann C, Orsini N, Pershagen G, Eller E, Kjaer HF, Gehring U, Granell R, Henderson J, Heinrich J, Lau S, Nieuwenhuijsen M, Sunyer J, Tischer C, Torrent M, Wahn U, Wijga AH, Wickman M, Keil T, Bergström A, ENRIECO Consortium (2012). Maternal smoking in pregnancy and asthma in preschool children: a pooled analysis of eight birth cohorts. Am J Respir Crit Care Med.

[4] Gorog K, Pattenden S, Antova T, Niciu E, Rudnai P, Scholtens S, Splichalova A, Slotova K, Vokó Z, Zlotkowska R, Houthuijs D (2011). Maternal smoking during pregnancy and childhood obesity: results from the CESAR Study. Matern Child Health J.

[5] O’Callaghan FV, Al Mamun A, O’Callaghan M, Alati R, Williams GM, Najman JM (2010). Is smoking in pregnancy an independent predictor of academic difficulties at 14 years of age? A birth cohort study. Early Hum Dev.

[6] Thapar A, Fowler T, Rice F, Scourfield J, van den Bree M, Thomas H, Harold G, Hay D (2003). Maternal smoking during pregnancy and attention deficit hyperactivity disorder symptoms in offspring. Am J Psychiatry.

[7] Huizink AC, Mulder EJ (2006). Maternal smoking, drinking or cannabis use during pregnancy and neurobehavioral and cognitive functioning in human offspring. Neurosci Biobehav Rev.

[8] Brook DW, Zhang C, Rosenberg G, Brook JS (2006). Maternal cigarette smoking during pregnancy and child aggressive behavior. Am J Addict.

[9] Batstra L, Hadders-Algra M, Neeleman J (2003). Effect of antenatal exposure to maternal smoking on behavioral problems and academic achievement in childhood: prospective evidence from a Dutch birth cohort. Early Hum Dev.

[10] Day NL, Richardson GA, Goldschmidt L, Cornelius MD (2000). Effects of prenatal tobacco exposure on preschoolers’ behavior. J Dev Behav Pediatr.

[11] Gilman SE, Gardener H, Buka SL (2008). Maternal smoking during pregnancy and children’s cognitive and physical development: a causal risk factor?. Am J Epidemiol.

[12] Batty GD, Der G, Deary IJ (2006). Effect of maternal smoking during pregnancy on offspring’s cognitive ability: empirical evidence for complete confounding in the US national longitudinal survey of youth. Pediatrics.

[13] Lambe M, Hultman C, Torrang A, Maccabe J, Cnattingius S (2006). Maternal smoking during pregnancy and school performance at age 15. Epidemiology.

[14] Wakschlag LS, Pickett KE, Cook E, Benowitz NL, Leventhal BL (2002). Maternal smoking during pregnancy and severe antisocial behavior in offspring: a review. Am J Public Health.

[15] Bublitz MH, Stroud LR (2012). Maternal smoking during pregnancy and offspring brain structure and function: review and agenda for future research. Nicotine Tob Res.

[16] Slotkin TA (2004). Cholinergic systems in brain development and disruption by neurotoxicants: nicotine, environmental tobacco smoke, organophosphates. Toxicol Appl Pharmacol.

[17] Roy TS, Seidler FJ, Slotkin TA (2002). Prenatal nicotine exposure evokes alterations of cell structure in hippocampus and somatosensory cortex. J Pharmacol Exp Ther.

[18] Thapar A, Rutter M (2009). Do prenatal risk factors cause psychiatric disorder? Be wary of causal claims. Br J Psychiatry.

[19] D’Onofrio BM, Lahey BB, Turkheimer E, Lichtenstein P (2013). Critical need for family-based, quasi-experimental designs in integrating genetic and social science research. Am J Public Health.

[20] Knopik VS (2009). Maternal smoking during pregnancy and child outcomes: real or spurious effect?. Dev Neuropsychol.

[21] Ellingson JM, Goodnight JA, Van Hulle CA, Waldman ID, D’Onofrio BM (2014). A sibling-comparison study of smoking during pregnancy and childhood psychological traits. Behav Genet.

[22] Ellingson JM, Rickert ME, Lichtenstein P, Langstrom N, D’Onofrio BM (2012). Disentangling the relationships between maternal smoking during pregnancy and co-occurring risk factors. Psychol Med.

[23] Agrawal A, Knopik VS, Pergadia ML, Waldron M, Bucholz KK, Martin NG, Heath AC, Madden PA (2008). Correlates of cigarette smoking during pregnancy and its genetic and environmental overlap with nicotine dependence. Nicotine Tob Res.

[24] Lahey BB, D’Onofrio BM (2010). All in the family: comparing siblings to test causal hypotheses regarding environmental influences on behavior. Curr Dir Psychol Sci.

[25] Rice F, Harold GT, Boivin J, Hay DF, van den Bree M, Thapar A (2009). Disentangling prenatal and inherited influences in humans with an experimental design. Proc Natl Acad Sci U S A.

[26] D’Onofrio BM, Rickert ME, Langstrom N, Donahue KL, Coyne CA, Larsson H, Ellingson JM, Van Hulle CA, Iliadou AN, Rathouz PJ, Lahey BB, Lichtenstein P (2012). Familial confounding of the association between maternal smoking during pregnancy and offspring substance use and problems. Arch Gen Psychiatry.

[27] Gaysina D, Fergusson DM, Leve LD, Horwood J, Reiss D, Shaw DS, Elam KK, Natsuaki MN, Neiderhiser JM, Harold GT (2013). Maternal smoking during pregnancy and offspring conduct problems: evidence from 3 independent genetically sensitive research designs. JAMA Psychiatry.

[28] Gilman SE, Loucks EB (2012). Invited commentary: does the childhood environment influence the association between every x and every y in adulthood?. Am J Epidemiol.

[29] Kaufman JS (2008). Commentary: why are we biased against bias?. Int J Epidemiol.

[30] Ashford J, van Lier PA, Timmermans M, Cuijpers P, Koot HM (2008). Prenatal smoking and internalizing and externalizing problems in children studied from childhood to late adolescence. J Am Acad Child Adolesc Psychiatry.

[31] Williams GM, O’Callaghan M, Najman JM, Bor W, Andersen MJ, Richards D (1998). C U. Maternal cigarette smoking and child psychiatric morbidity: a longitudinal study. Pediatrics.

[32] Brion MJ, Victora C, Matijasevich A, Horta B, Anselmi L, Steer C, Menezes AM, Lawlor DA, Davey SG (2010). Maternal smoking and child psychological problems: disentangling causal and noncausal effects. Pediatrics.

[33] Carter S, Paterson J, Gao W, Iusitini L (2008). Maternal smoking during pregnancy and behavior problems in a birth cohort of 2-year-old Pacific children in New Zealand. Early Hum Dev.

[34] Robinson M, McLean NJ, Oddy WH, Mattes E, Bulsara M, Li J, Zubrick SR, Stanley FJ, Newnham JP (2010). Smoking cessation in pregnancy and the risk of child behavioral problems: a longitudinal prospective cohort study. J Epidemiol Community Health.

[35] Murray CJ, Vos T, Lozano R, Naghavi M, Flaxman AD, Michaud C, Ezzati M, Shibuya K, Salomon JA, Abdalla S, Aboyans V, Abraham J, Ackerman I, Aggarwal R, Ahn SY, Ali MK, Alvarado M, Anderson HR, Anderson LM, Andrews KG, Atkinson C, Baddour LM, Bahalim AN, Barker-Collo S, Barrero LH, Bartels DH, Basáñez MG, Baxter A, Bell ML, Benjamin EJ (2012). Disability-adjusted life years (DALYs) for 291 diseases and injuries in 21 regions, 1990–2010: a systematic analysis for the Global Burden of Disease Study 2010. Lancet.

[36] Magnus P, Irgens LM, Haug K, Nystad W, Skjaerven R, Stoltenberg C (2006). Cohort profile: the Norwegian Mother and Child Cohort Study (MoBa). Int J Epidemiol.

[37] Achenbach TM (1992). Manual for the Child Behavior Checklist/2-3 and 1992 Profile.

[38] Clark LA, Watson D (1995). Constructing validity: basic issues in objective scale development. Psychol Assess.

[39] Novik TS (1999). Validity of the child behavior checklist in a Norwegian sample. Eur Child Adolesc Psychiatry.

[40] Novik TS (2000). Child behavior checklist item scores in Norwegian children. Eur Child Adolesc Psychiatry.

[41] Strand BH, Dalgard OS, Tambs K, Rognerud M (2003). Measuring the mental health status of the Norwegian population: a comparison of the instruments SCL-25, SCL-10, SCL-5 and MHI-5 (SF-36). Nord J Psychiatry.

[42] Tambs K, Moum T (1993). How well can a few questionnaire items indicate anxiety and depression?. Acta Psychiatr Scand.

[43] Fitzmaurice GM, Laird NM, Ware JH (2004). Applied Longitudinal Analysis.

[44] Keshavan MS, Murray R (1997). Neurodevelopment and Adult Psychopathology.

[45] Cohen J, Cohen J (2003). Applied Multiple Regression/Correlation Analysis for the Behavioral Sciences.

[46] Roza SJ, Verhulst FC, Jaddoe VW, Steegers EA, Mackenbach JP, Hofman A (2009). Maternal smoking during pregnancy and child behavior problems: the Generation R Study. Int J Epidemiol.

[47] Lavigne JV, Hopkins J, Gouze KR, Bryant FB, LeBailly SA, Binns HJ (2011). Is smoking during pregnancy a risk factor for psychopathology in young children? A methodological caveat and report on preschoolers. J Pediatr Psychol.

[48] Orlebeke JF, Knol DL, Verhulst FC (1997). Increase in child behavior problems resulting from maternal smoking during pregnancy. Arch Environ Health.

[49] Abbott LC, Winzer-Serhan UH (2012). Smoking during pregnancy: lessons learned from epidemiological studies and experimental studies using animal models. Crit Rev Toxicol.

[50] DeBry SC, Tiffany ST (2008). Tobacco-induced neurotoxicity of adolescent cognitive development (TINACD): a proposed model for the development of impulsivity in nicotine dependence. Nicotine Tob Res.

[51] Roy TS, Sabherwal U (1994). Effects of prenatal nicotine exposure on the morphogenesis of somatosensory cortex. Neurotoxicol Teratol.

[52] Eppolito AK, Bachus SE, McDonald CG, Meador-Woodruff JH, Smith RF (2010). Late emerging effects of prenatal and early postnatal nicotine exposure on the cholinergic system and anxiety-like behavior. Neurotoxicol Teratol.

[53] Moylan S, Jacka FN, Pasco JA, Berk M (2013). How cigarette smoking may increase the risk of anxiety symptoms and anxiety disorders: a critical review of biological pathways. Brain Behav.

[54] Pickett KE, Rathouz PJ, Kasza K, Wakschlag LS, Wright R (2005). Self-reported smoking, cotinine levels, and patterns of smoking in pregnancy. Paediatr Perinat Epidemiol.

[55] Najdawi F, Faouri M (1999). Maternal smoking and breastfeeding. East Mediterr Health J.

[56] Gustavson K, von Soest T, Karevold E, Roysamb E (2012). Attrition and generalizability in longitudinal studies: findings from a 15-year population-based study and a Monte Carlo simulation study. BMC Public Health.

[57] Nilsen RM, Vollset SE, Gjessing HK, Skjaerven R, Melve KK, Schreuder P, Alsaker ER, Haug K, Daltveit AK, Magnus P (2009). Self-selection and bias in a large prospective pregnancy cohort in Norway. Paediatr Perinat Epidemiol.

